# Efficient Determination of Slip-Link Parameters from Broadly Polydisperse Linear Melts

**DOI:** 10.3390/polym10080908

**Published:** 2018-08-12

**Authors:** Néstor E. Valadez-Pérez, Konstantin Taletskiy, Jay D. Schieber, Maksim Shivokhin

**Affiliations:** 1 Department of Chemical and Biological Engineering and Center for Molecular Study of Condensed Soft Matter, Illinois Institute of Technology, 3440 S. Dearborn Street, Chicago, IL 60616, USA; ktaletsk@hawk.iit.edu; 2 Department of Physics and Department of Applied Mathematics, Illinois Institute of Technology, 3101 S. Deaborn St., Chicago, IL 60616, USA; 3 ExxonMobil Chemical Company, 5200 Bayway Drive, Baytown, TX 77520, USA; maksim.e.shivokhin@exxonmobil.com

**Keywords:** polymer melts, entanglements, rheology, simulations

## Abstract

We investigate the ability of a coarse-grained slip-link model and a simple double reptation model to describe the linear rheology of polydisperse linear polymer melts. Our slip-link model is a well-defined mathematical object that can describe the equilibrium dynamics and non-linear rheology of flexible polymer melts with arbitrary polydispersity and architecture with a minimum of inputs: the molecular weight of a Kuhn step, the entanglement activity, and Kuhn step friction. However, this detailed model is computationally expensive, so we also examine predictions of the cheaper double reptation model, which is restricted to only linear rheology near the terminal zone. We report the storage and loss moduli for polydisperse polymer melts and compare with experimental measurements from small amplitude oscillatory shear. We examine three chemistries: polybutadiene, polypropylene and polyethylene. We also use a simple double reptation model to estimate parameters for the slip-link model and examine under which circumstances this simplified model works. The computational implementation of the slip-link model is accelerated with the help of graphics processing units, which allow us to simulate in parallel large ensembles made of up to 50,000 chains. We show that our simulation can predict the dynamic moduli for highly entangled polymer melts over nine decades of frequency. Although the double reptation model performs well only near the terminal zone, it does provide a convenient and inexpensive way to estimate the entanglement parameter for the slip-link model from polydisperse data.

## 1. Introduction

The dynamic modulus G* of polymer melts is a standard, and relatively easy rheological measurement, useful for characterizing polymer melt blends, and is sensitive to chain architecture and molecular weight [1]. For example, star-branched architectures show a much stronger dependence on molecular weight than do linear chains, and a particular shape near the crossover regime. Hence, it would be useful to develop tools to extract information about molecular weight distributions and branching from G* using a reliable molecular model.

Conceptually, at least, there is a forward problem—predicting rheology from a known dispersity of structure and molecular weight—and a backward problem—predicting the dispersity from a measured dynamic modulus. Ideally, one would like to have a reliable, fast, cheap tool that solves the backward problem. To develop such a tool, however, requires a good theoretical framework for the forward problem, which is our focus here. We also restrict ourselves to linear chains of polydisperse molecular weight distribution. The forward problem is typically handled using a tube picture. Unfortunately, recent experiments on blends of monodisperse linear and monodisperse star-branched chains have shown that inexpensive tube models are unable to describe such data reliably [2]. On the other hand, the more costly discrete slip-link model (DSM) was able to make quantitative predictions. Moreover, the slip-link model is also appropriate for predicting nonlinear rheology, without parameter adjustment, for both linear and branched polymers.

The dynamic behavior of polymer chains in a large enough concentration melt or suspension depends on the topological constrictions imposed on one chain arising from the chains in the matrix. Such physics is often represented by a polymer chain moving or reptating inside a virtual tube made for all the constrictions. This tube model was originally proposed by de Gennes [3] and developed by Doi and Edwards [4]. In the first version of the model, both the diameter of the tube and the portion of chain inside the tube were kept constant. Later models include other dynamical mechanisms. For example, the tube can reorganize, which allows additional relaxation—a process known as constraint-release [5]—and the possibility of changing the span of the random-walk chain inside the tube, known as contour-length fluctuation [6]. A well-known model that includes the constraint-release mechanism is double reptation [7,8,9,10]. This model assumes an entanglement made by two chains can disappear by the reptation of either chain, and is particularly useful to study polydisperse systems [11,12].

Besides tube models, there are computational models to study the relaxation of chains without involving the concept of a tube. For example, the direct simulation of chains, made out of a certain number of interconnected monomers, has been carried out through molecular dynamics simulations [13,14,15]. A different approach is used by slip-link models in which entanglements are discrete objects [16,17,18,19,20,21]. Those models take into account the explicit creation and destruction of entanglements along polymer backbones in a stochastic way described by a master equation. Recently, some of us developed an algorithm to calculate the dynamic modulus of polydisperse systems relatively efficiently [22]. Rather than simulating a large number of molecular weights, one simulates a small number of probe chains, and then interpolates between them to integrate over the molecular weight distribution. Since each probe chain sees the same dynamic background for constraint dynamics, additional probes can be added as needed to improve the prediction, without requiring new calculations of the prior probes.

Here, we examine in detail the ability of the slip-link model to make quantitative predictions of industrial, polydisperse blends. However, many distributions of industrial interest have chains containing several hundred entanglements, on average, which is particularly expensive for DSM. Therefore, we also examine a simpler and cheaper double-reptation model (DRM). Linear DRM might be expected to perform reasonably well for chains of relatively broad width, but not for nearly monodisperse systems, where DSM is straightforward to calculate. Hence, we also examine mapping the parameters of these two complementary models onto each other, so that they might be used somewhat interchangeably. We performed calculations for polydisperse 1,4-polybutadiene (PBD), polypropylene (PP) and polyethylene (PE) melts and compare our predictions with experimental results.

We found that the modulus parameter of the double reptation model can be used to estimate the entanglement parameters of DSM. On the other hand, we found that the uncertainty in DRM parameters are too large to allow a reasonable estimate of the DSM frictional parameter. This limitation is not particularly problematic, however, since all calculations are performed without dimensions, which eliminates friction.

In both DRM and DSM approaches here, we describe molecular weight distributions W(M) by the so-called generalized exponential (GEX) function, whose parameters are determined by gel permeation chromatography (GPC) data. The models or calculations are made somewhat simpler by using such an analytic molar mass distribution, but it is not necessary. Even a numerical distribution could be used. In addition, the DRM requires two parameters: the plateau modulus, $G_{N}^{0}$, and a characteristic time, $\tau_{0}$. From a direct comparison with dynamic modulus data, we obtain their values.

In Section 2, we describe the experimental samples we used in our study. In Section 3, we review some elements of the double reptation model and show the calculation for the parameters that characterize our polymer melts. In Section 4, we present the key elements of the CFSM and their relation with the DRM. Finally, in Section 5, we present the conclusions of the present work.

## 2. Experiments

### 2.1. Samples

In this study, we examined three kinds of industrial linear polymers: atactic polypropylene (PP), polyethylene (PE) and 1,4-polybutadiene (PBD). All samples are characterized by a similar polydispersity index (PDI), nominally 2. PP and PE samples were produced and analyzed in the ExxonMobil facilities, whereas the data for the PBD samples were published in Ref. [23]. In Table 1, we present the weight-average molecular weight ($M_{w}$), polydispersity index ($PDI_{1}$) and the temperature (*T*) at which rheological measurements are reported. We also include an estimate for the plateau modulus parameter ($G_{N}^{0}$, related to but not identical with the observed plateau in dynamic modulus data) made by using a tube model, the density (ρ), and molecular weight of a Kuhn step ($M_{K}$) reported in Ref. [24]. The PP and PBD samples have very different average molecular weight compared with those of the PE samples.

### 2.2. Characterization

**Figure 1:**
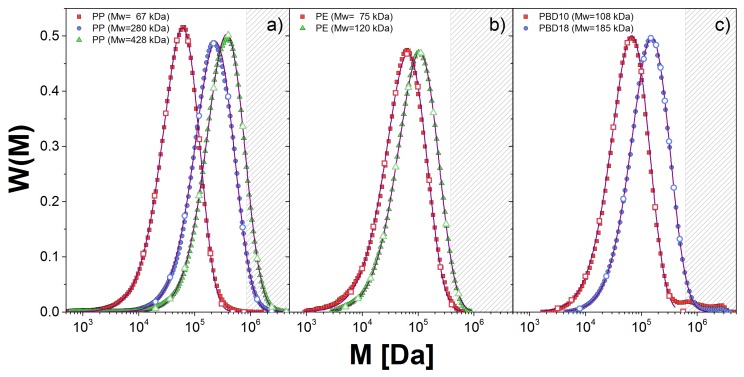
Molecular weight distribution, W(M), obtained from GPC measurements (symbols) for: (**a**) PP; (**b**) PE; and (**c**) PBD melts. The fitting to the GEX distribution is shown with solid lines. The discrete distribution used in the DSM approach is given by the empty symbols.

To characterize the molecular weight distribution, W(M), of the samples we used gel permeation chromatography (GPC). In Figure 1, we show W(M) (described below) for the linear polymer melts (solid lines) along with the corresponding fitting to the generalized exponential distribution, GEX (dashed lines). This distribution is used for convenience since the GEX parameters are used as inputs for the two theoretical approaches used in this work, as we explain below.

The GEX function is given by

(1) $$W\left(M\right)=\frac{b}{\Gamma \left(\frac{a+1}{b}\right)}{\left(\frac{M}{m_{p}}\right)}^{a+1}\exp\left[-{\left(\frac{M}{m_{p}}\right)}^{b}\right],$$

where the parameters *a* and *b* determine the shape of the distribution and $m_{p}$ is related with the localization of the distribution center [25]. Γ(z) is the gamma function defined for any z>0. This mass distribution is weighted by chain mass, and is normalized by the weight-averaged molecular weight

(2) $$\int_{0}^{\infty }W(M)dM=M_{w},$$

see Table 1. There is significant variation in notation and definition in the literature about the molecular weight distribution, including a quantity equivalent to $\frac{dW(M)}{d\log_{10}M}$, which can result in a factor of ln10 in integration [23]. The molar mass averages can be related to the GEX parameters [25]

(3) $$M_{n}=m_{p}\frac{\Gamma \left(\frac{a+1}{b}\right)}{\Gamma \left(\frac{a}{b}\right)},\;M_{w}=m_{p}\frac{\Gamma \left(\frac{a+2}{b}\right)}{\Gamma \left(\frac{a+1}{b}\right)},\;M_{z}=m_{p}\frac{\Gamma \left(\frac{a+3}{b}\right)}{\Gamma \left(\frac{a+2}{b}\right)},$$

with $M_{n}<M_{w}<M_{z}$. Then, the polydispersity indices are

(4) $$PDI_{1}:=\frac{M_{w}}{M_{n}},\;\;PDI_{2}:=\frac{M_{z}}{M_{w}}.$$

For unimodal distributions, $PDI_{2}<PDI_{1}$. However, if W(M) contains small traces of high weight polymer chains, $PDI_{2}$ could be larger than $PDI_{1}$.

We show in Table 2 the values for the GEX parameters for all samples, except for PBD20 which is considered nearly monodisperse. The error bars for the estimation of *a* and *b* are small, but that for $m_{p}$ is large, which presents some limitations discussed below. Nonetheless, our fitting captures W(M) well near the maximum of the distribution in all cases. However, for PBD samples, we observe small shoulders at high weights which were discarded to perform the fitting. The neglect of these shoulders in estimating the dynamic modulus is visible at the lowest frequencies below.

### 2.3. Rheological Measurements

Dynamic and loss moduli, $G^{\prime }\left(\omega \right)$ and $G^{\prime \prime }\left(\omega \right)$, respectively, were determined through small amplitude oscillatory shear (SAOS) experiments. The measurements were performed at 120 and 80 ${}^{\circ }$C for the PP and at 190 ${}^{\circ }$C for the PE.

## 3. Double Reptation Model

It is well established that the mechanical properties of polymers melts are determined by their chemical composition, molecular weight and polydispersity [2]. In this theoretical study, we focus on the effect of the molecular weight distribution while the effect of the chemistry is included in the parameters. We follow the approach called the double reptation model (DRM), developed by des Cloizeaux [8] and Tsenoglou [10].

For DRM, the relaxation modulus for a linear melt, whose polydispersity is estimated by W(M), is given by [10]

(5) $$G\left(t\right)=G_{N}^{0}{\left(\int_{M_{crit}}^{\infty }W\left(M\right)\sqrt{\exp\left(-\frac{t}{\tau (M)}\right)}\frac{dM}{M}\right)}^{2}$$

where $M_{crit}$ is a critical molecular mass and τ is a characteristic relaxation time for a polymer with a molecular mass *M*. This modulus can also be expressed in terms of the relaxation spectrum, h(τ),

(6) $$G\left(t\right)=\int_{0}^{\infty }h\left(\tau \right)\exp\left(-t/\tau \right)\frac{d\tau }{\tau }.$$

To relate the previous equations, the DRM assume that each molecular weight contributes only a single relaxation time to the spectrum and we use the relation proposed in Ref. [10],

(7) $$\tau \left(M\right)=kM^{\alpha },$$

where the value of the exponent α is set to 3.5, consistent with the observed scaling of the longest relaxation time with molecular weight for monodisperse linear melts and *k* is a chemistry- and temperature-dependent parameter [10]. It is also assumed that the effects of sliding dynamics and constraint dynamics can be factorized in the time domain, and that these functions are a single exponential decay, identical to one another. As a result, Thimm et al. [26] calculated the relaxation spectrum as

(8) $$\tilde{h}\left(M\right)=\frac{2G_{N}^{0(DRM)}}{\alpha }W\left(M\right)\left(\int_{M}^{\infty }W\left(M^{\prime }\right)\frac{dM^{\prime }}{M^{\prime }}\right),$$

where $\tilde{h}\left(M\right)$ is the relaxation spectrum as a function of molecular weight: $\tilde{h}\left(M\right)=h\left(\tau \left(M\right)\right)$. Here, we point out that the plateau modulus parameter, $G_{N}^{0(DRM)}$, obtained for this model could be different from that obtained with another theoretical or experimental approach.

As often done (e.g., in the work of Guzman et al. [25]), the storage and loss moduli for polydisperse melts can be recast in terms of h(τ) [1] as

(9) $$\begin{array}{c} G^{\prime }\left(\omega \right)=\int_{0}^{\infty }h\left(\tau \right)\frac{{\left(\omega \tau \right)}^{2}}{1+{\left(\omega \tau \right)}^{2}}\frac{d\tau }{\tau }, \end{array}$$

(10) $$\begin{array}{c} G^{\prime \prime }\left(\omega \right)=\int_{0}^{\infty }h\left(\tau \right)\frac{\omega \tau }{1+{\left(\omega \tau \right)}^{2}}\frac{d\tau }{\tau }, \end{array}$$

by taking the Laplace–Fourier transform of Equation (6).

If we assume a GEX MWD, then using Equation (1) in Equation (8) the spectrum is expressed in the compact form

(11) $$\tilde{h}\left(M\right)=2G_{N}^{0(DRM)}W\left(M\right)\frac{\Gamma \left(\frac{1+a}{b},{\left(\frac{M}{m_{p}}\right)}^{b}\right)}{\alpha \Gamma \left(\frac{1+a}{b}\right)},$$

where Γ(z,x) is the incomplete gamma function. Inserting this equation into Equations (9) and (10), we can obtain the complex modulus via numerical integration. Introducing the dimensionless quantities $N=M/m_{p}$, $\tau_{0}=km_{p}^{\alpha }$ and $\Omega =\omega \tau_{0}$, and defining the dimensionless relaxation spectrum as $H\left(N\right)=\alpha \tilde{h}\left(m_{p}N\right)/G_{N}^{0}$, the complex modulus is expressed as

(12) $$\begin{array}{c} G^{\prime }\left(\omega \right)=G_{N}^{0}\int_{0}^{\infty }H\left(N\right)\frac{{\left(\Omega N^{\alpha }\right)}^{2}}{1+{\left(\Omega N^{\alpha }\right)}^{2}}\frac{dN}{N}, \end{array}$$

(13) $$\begin{array}{c} G^{\prime \prime }\left(\omega \right)=G_{N}^{0}\int_{0}^{\infty }H\left(N\right)\frac{\Omega N^{\alpha }}{1+{\left(\Omega N^{\alpha }\right)}^{2}}\frac{dN}{N}. \end{array}$$

A detailed derivation of the previous equations can be found in Ref. [25]. The model depends on two rheology parameters, $G_{N}^{0(DRM)}$ and *k*, which are obtained by a direct comparison with experimental measurements of G* in two steps.

**Figure 2:**
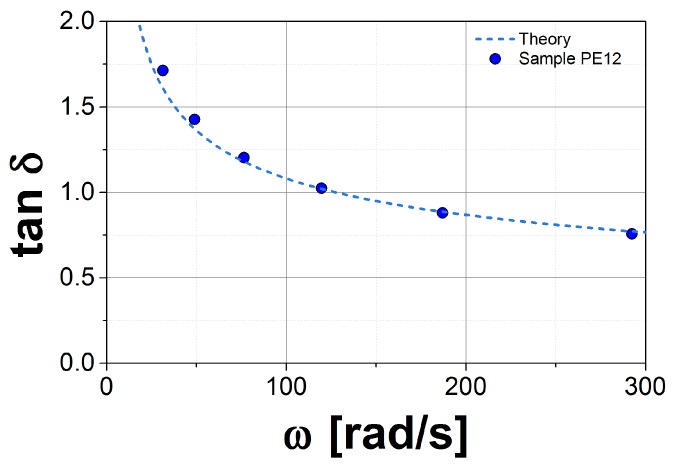
Comparison between the tan(δ) for experiments (symbols) and theory (dashed lines) for the sample PE12.

In the first step, we plot the loss tangent, $\tan\delta =G^{\prime \prime }/G^{\prime }$, as a function of frequency, ω, for both the experimental data and the theory. This function is independent of $G_{N}^{0}$, so we can estimate $\tau_{0}$, or equivalently *k*, by matching the theoretical prediction to the experimental data in the vicinity of the crossover point (CP), tanδ=1 and $\omega =\omega_{\mathrm{CP}}$. In Figure 2, we show this plot for sample PE12. This procedure eliminates uncertainties in $G_{N}^{0(DRM)}$ from fitting *k*. The second step consists in matching the theory to the experimental data in the G* axis, again in the vicinity of the crossover point. By using this method, $G_{N}^{0}$ is well defined for this model, but the obtained values may not be in agreement with the conventional methods used in other tube models (see, for example, Ref. [27]).

As mentioned in Ref. [27], there are various ways to define the value of the plateau modulus for monodisperse samples, PDI∼1, with high molecular weights. However, obtaining this value in polydisperse samples is more difficult, even though the actual value is presumed independent of the polydispersity [28,29,30].

### 3.1. Results for the DRM

**Figure 3:**
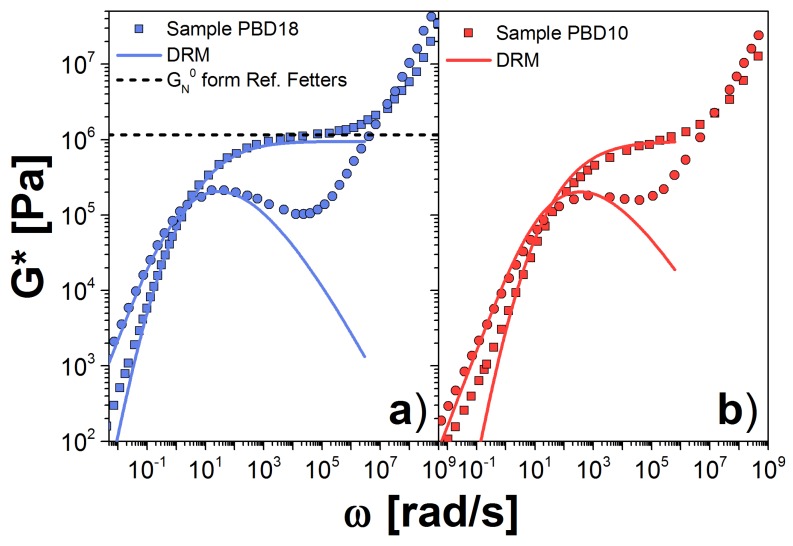
Dynamic moduli for PBD samples: (**a**) PBD18; and (**b**) PBD10 at 30 ${}^{\circ }$C. Symbols are the experimental results reported in Ref. [23]; solid lines are our theoretical prediction using the DRM; and the dashed line is the plateau modulus reported in Ref. [24]. Squares are storage modulus, $G^{\prime }$, and circles are loss modulus, $G^{\prime \prime }$. Our estimations for the parameters $G_{N}^{0}$ and $\tau_{0}$ are listed in Table 2. In panel (**b**), we used the $G_{N}^{0}$ obtained for the PBD18 sample.

First, we analyze PBD, since this polymer melt has been studied extensively in the literature [2,23,31,32,33]. In Figure 3, we plot our results for (Figure 3a) PBD18 and (Figure 3b) PBD10 samples at 30 ${}^{\circ }$C, along with the experimental data from Ref. [23]. For both samples, the theoretical prediction fits the experiments very well at frequencies near the crossover point (CP), as expected. At small frequencies, $\omega \ll \omega_{\mathrm{CP}}$, the theory reproduces the experimental behavior of PBD18 but not of PBD10. This discrepancy is because the MWD of PBD18 is well represented by the GEX distribution. However for PBD10 the W(M) contains a small tail due to the presence of high molecular chains, not included in the GEX expression. Such high–molecular–weight chains have large relaxation times, associated with the low frequencies discrepancy.

For frequencies above $\omega_{\mathrm{CP}}$, $G^{\prime }$ increases until it reaches the plateau value, $G_{N}^{0(DRM)}$ while $G^{\prime \prime }$ falls below the data as ω increases. This discrepancy between model and experiment in the loss modulus near the plateau presumably arises from the assumption of a single relaxation time for each molecular weight. We might be able to improve our estimation by including the contribution of the Rouse modes to G*, as is done in Ref. [25].

For PBD18, we estimated $G_{N}^{0(DRM)}=0.948$ MPa, this value is smaller than $G_{0}^{N}=1.15$ MPa, reported in Ref. [24] and used to model PBD in Ref. [23], see the dashed line in Figure 3a. For the other PBD sample $G_{N}^{0(DRM)}=0.690$ MPa, again the modulus is different from that reported in Ref. [24]. In both cases, the estimation of *k* results in a very similar value, since *k* depends on the chemistry and the temperature but not on the molecular weight of the polymer. However, $\tau_{0}$ is very different in both cases, since it depends on the calculation of $m_{p}$, which cannot be determined accurately for any of the polymers we analyzed. Table 2 summarizes all of the values for $G_{N}^{0(DRM)}$, *k* and $\tau_{0}$ corresponding to all the polymer samples in this work.

As a check, we use the $G_{N}^{0}$ value obtained from the PBD18 sample to calculate G* for the sample PBD10, shown in Figure 3b. Again, we observe good agreement between theory and experiment, suggesting consistency between the samples, and between the data and the theory. However, we found an important deviation in the terminal zone, which is due to large chains in the sample, represented as a shoulder in the W(M). The high molecular weight contribution is larger in the PBD10 sample than in the PBD18 sample.

**Figure 4:**
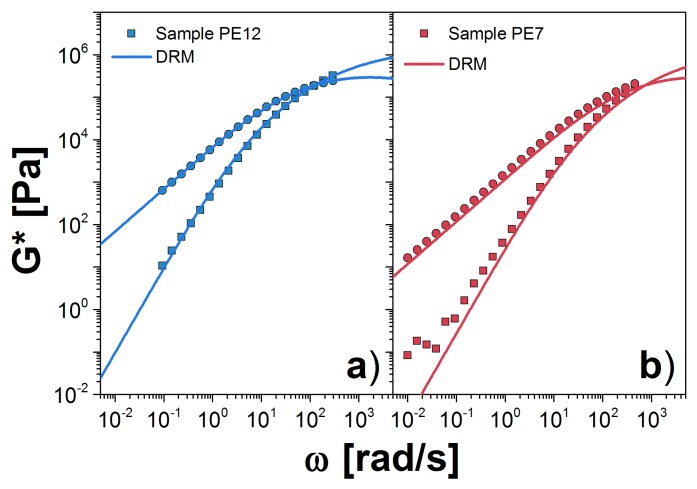
Dynamic moduli for PE samples: (**a**) PE12; and (**b**) PE7 at $190^{\circ }$C (solid symbols). Our prediction using the DRM is represented by solid lines. The corresponding values for $G_{N}^{0}$ and $\tau_{0}$ are shown in Table 2. In panel (**b**), we used the $G_{N}^{0}$ obtained for the PE12 sample.

Next, we analyze the PE samples at 190 ${}^{\circ }$C. We consider first the melt with the largest $M_{w}$, PE12. For this melt, we obtained $G_{N}^{0(DRM)}=1.44$ MPa and $k=1.02\times 10^{-20}$ s(mol/g)${}^{3.5}$. This plateau is almost one half of that reported by Fetter et al. [24]. However, it is an acceptable value as shown in Ref. [27]. We observe excellent agreement between theory and experiment for all accessible frequencies in the experiment, as shown in Figure 4a. For PE7, we obtained $G_{N}^{0(DRM)}=1.89$ MPa and $k=0.958\times 10^{-20}$ s(mol/g)${}^{3.5}$. The value for *k* is very similar to that for PE12, which corroborates the independence of *k* on molecular weight. However, the plateau modulus is more than 30% larger, which probably arises from uncertainty in extrapolation to the plateau from data that exist only in the terminal zone. In addition, there is typically a variation of approximately 20% in the observed plateau from sample to sample, owing to its strong dependence on sample radius when using parallel plates in the rheometer [21]. In Figure 4b, we plot G* for PE7 using the modulus value fit to PE12 data. Agreement with the experimental data is good.

**Figure 5:**
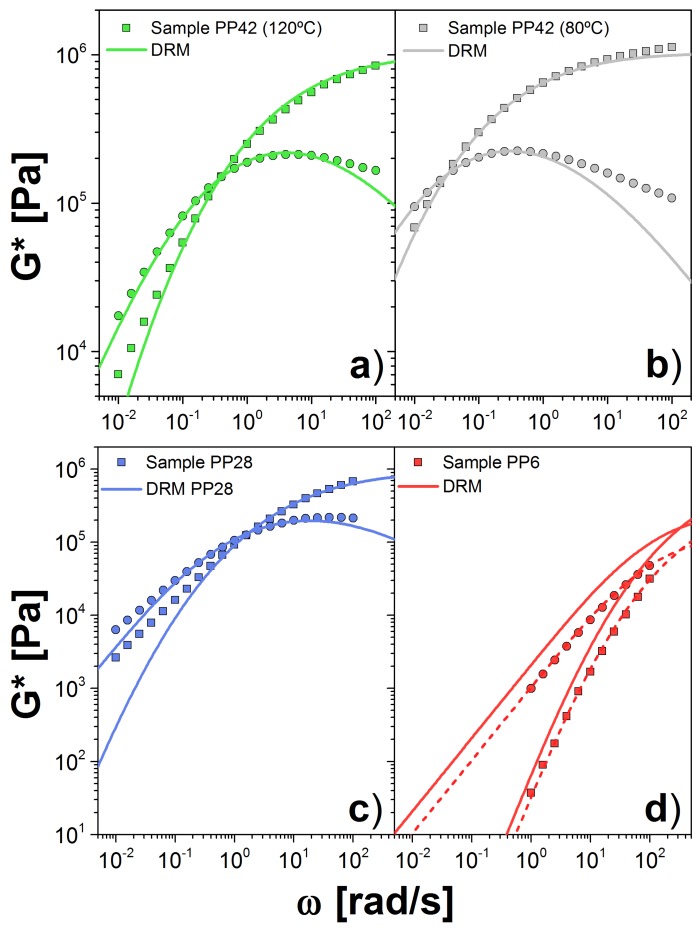
Dynamic moduli for PP samples PP42 at: (**a**) 120 ${}^{\circ }$C; and (**b**) 180 ${}^{\circ }$C; and for: (**c**) PP28; and (**d**) PP6 at 120 ${}^{\circ }$C. Symbols are the experimental data (squares = $G^{\prime }$, circles = $G^{\prime \prime }$) and solid lines are the theoretical prediction using the DRM. In (**d**), we include the calculation using the $G_{N}^{0(DRM)}$ obtained for the PP42 sample (solid line) and PP6 sample (dashed line).

The last dataset is from the PP melts. Sample PP42 was measured at both 80 and 120 ${}^{\circ }$C, and the others only at 120 ${}^{\circ }$C. The largest $M_{w}$, PP42, is the most entangled sample we analyzed. Our calculation showed that $G_{N}^{0(DRM)}=1.000\left(1.025\right)$ MPa and $k=4.51\left(59.42\right)\times 10^{-20}$ s(mol/g)${}^{3.5}$ for the sample at 120(80) ${}^{\circ }$C. This is twice the value obtained for the modulus calculated by other approaches [24,34]. Good agreement with the experimental data is shown in Figure 5a,b, aside from the discrepancies at high frequency, explained above. The value of the plateau modulus, computed with this approach, varies slightly with temperature. On the other hand, *k* changes dramatically with temperature, more than expected.

For PP28, we observed a similar value for the plateau, $G_{N}^{0(DRM)}=0.918$ MPa, with respect to the PP42 sample. However, for the low $M_{w}$ sample, PP6, the plateau is considerably lower, $G_{N}^{0(DRM)}=0.47$ MPa. On the other hand, for both samples, we obtained a similar value for *k*: 4.54 and $k=3.99\times 10^{-20}$ s(mol/g)${}^{3.5}$ for PP28 and PP6, respectively. In Figure 5c,d, we show the comparison between experiments and theory for G*. In the case of PP6, we show the curves using the fit plateau value (dashed lines) as well as the plateau obtained from the PP42 sample (solid lines). Given the factor of 2 difference in the fitted plateau moduli, the vertical displacement between the two curves is not surprising. Unlike the two chemistries above, there appears to be a lack of consistency between the two samples.

Even though the DRM oversimplifies the features of a polymer melt, it is nonetheless able to describe the rheological behavior of melts for frequencies in the terminal zone and near the crossover. To have a complete picture of G* at high frequencies, we use the DSM below. One of the parameters in this model, β, determines the value for the plateau modulus, which is not easy to extract from polydisperse systems. Below, we examine whether the modulus parameter found from the double reptation model can be used to estimate the value for β in the detailed molecular model.

## 4. Clustered Fixed-Slip-Link Model

The DSM is a single-chain mean-field mathematical model proposed by Schieber et al. to study the equilibrium and non-equilibrium properties of polymers [16], and developed in later works [17,18,19]. The probe chain is modeled as a random walk of Kuhn steps forming Z(t)−1 entanglements with background chains. Entanglements are randomly distributed along the path, whose average number is fixed and related to the parameter β. This model has three chemistry-dependent parameters: the molecular weight of a Kuhn step ($M_{K}$), the entanglement activity (β) and the Kuhn step shuffling characteristic time ($\tau_{K}$). $M_{K}$ is determined by the chemistry of the polymer and it is not adjustable, since it can be found by independent means. The entanglement activity β is related to the value of the plateau modulus and $\tau_{K}$ is obtained from a direct comparison with experimental results of rheology, in the same way we obtained $\tau_{0}$ for the DRM.

### 4.1. The Parameters

In the DSM approach we simulate a main probe with different number of entanglements, on average, that depends on the molecular weight of the chain. Chains in the high-molecular-weight tail, of the corresponding W(M), have a huge number of entanglements which makes their simulation intractable using the original DSM. Intead, we use a coarse-grained version called the clustered fixed slip-link model (CFSM) developed by Andreev et al. [20]. In this model, Kuhn steps are clustered with a mass is given by

(14) $$M_{c}=0.56M_{K}\left(\beta +1\right).$$

Now, the number of clusters in a chain with a molecular mass $M_{w}$ is given by $N_{c}=M_{w}/M_{c}$. This value can be estimated from the high-frequency plateau, $G_{N}^{0}$, by solving the equation

(15) $$G_{N}^{0}=\frac{\rho RT}{M_{w}}\left[{\left(\frac{1}{2}\right)}^{N_{c}-1}+\frac{N_{c}-3}{2}\right],$$

where *R* is the ideal gas constant; ρ is the density; *T* is the absolute temperature; and $G_{N}^{0}$ is the plateau modulus, closely related to but not identical with the experimentally observed plateau value. We calculate the $N_{c}$ by solving Equation (15), and also we compute the corresponding $M_{c}$. Then, we calculate the value of β using Equation (14). We have to point out that the plateau modulus is an ambiguous quantity that could depend on the model used, the technique used to extract the rheological data and the conditions at which the measurements were performed. The most reliable value for the plateau modulus is obtained from rheological data obtained for a high molecular and monodisperse sample [18]. To obtain a consistent data, we need such parameter for the rest of polymers however such information could not be available. Instead, we use the $G_{N}^{0}$ obtained from our fitting to rheological data using the DRM, to estimate the entanglement density.

The characteristic time for a cluster is related with the characteristic time for a Kuhn step as

(16) $$\tau_{c}\approx 0.265\tau_{K}\beta^{8/3}.$$

Since the discrete slip-link model is mean field, the only influence on the relaxation of a single chain is through constraint dynamics (CD). As described in detail elsewhere [20], upon creation each entanglement is assigned a characteristic CD time determined by its environment.

### 4.2. Implementation

Calculations for this model are made using a stochastic numerical algorithm described elsewhere for the creation and destruction of entanglements along the path of the probe chain by two processes: sliding dynamics (SD) and constraint dynamics (CD). SD is responsible for the formation/creation of entanglements at the ends of the probe chain. Such entanglements connect the probe chain with the background chains. The CD refers to the formation/creation of entanglements by the background chains, with the probe chain, through the matrix chains’ SD. More details about this process can be found in Refs. [17,35].

The relaxation modulus is found using the Green–Kubo expression [36]

(17) $$G\left(\tau \right)=\frac{1}{n_{c}k_{B}T}{\langle \tau_{xy}\left(t\right)\tau_{xy}\left(t+\tau \right)\rangle }_{\mathrm{eq}},$$

where ${\langle \rangle }_{\mathrm{eq}}$ indicates the time or ensemble average at equilibrium conditions, $\tau {\left(t\right)}_{xy}$ is the stress tensor and $n_{c}$ is the number of chains per unit of volume. These calculations are much more expensive than DRM calculations, and grow with entanglement number to the 4.5 power. Hence, very highly entangled chains are prohibitive. However, the predictions are much better for the entire frequency range, unlike DRM. The study of branched architectures is also possible by using the DSM approach, but the calculations are even more expensive [2].

In a recent work, the DSM was used to study the mechanical properties of a cross-linked polymer [35], with an entangled solvent. In that work, the polydisperse polymer melt solvent was represented as the blend of two monodisperse melts whose first and second moment reproduce those of the original W(M). The relaxation modulus for each component was obtained and appropriately weighted, according to the corresponding W(M), to calculate the dynamic modulus of the polydisperse melt. This bidisperse approximation worked for melts with a $PDI_{1}\leq 1.61$.

In this work, we are dealing with a broad polydisperse melt so we use the same approach as in Ref. [35]. In our case, the continuous distribution is represented by several probe chains (order 10), whose molecular weights, $\left\{M_{i}\right\}$, are consistent with the GEX distribution. These probes then allow us to integrate the molecular weight at each frequency numerically by cubic spline interpolation. This approach allows us to have a better representation of the molecular weight distribution, but increases the computational cost of the calculations.

**Figure 6:**
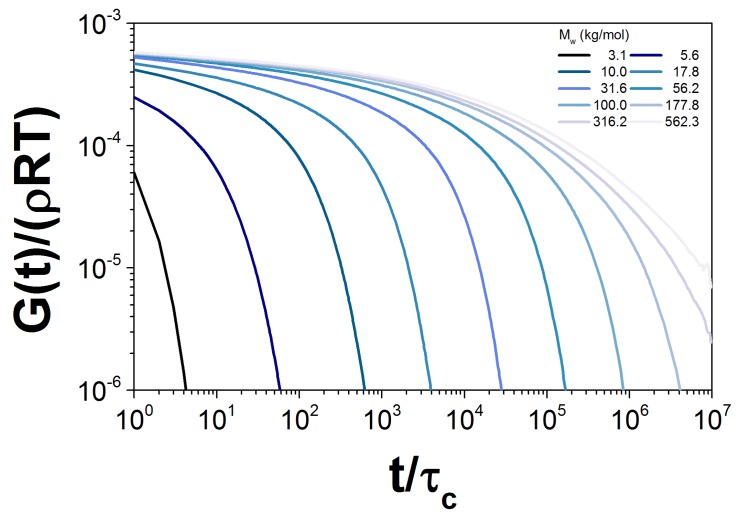
Normalized G(t) for the simulation of ten probes with different $M_{w}$, corresponding to the PBD10 sample.

To calculate G(t) for every probe, we simulated an ensemble of 5000 chains. The probe weights chosen are shown as open symbols in Figure 1. To illustrate, in Figure 6, we show the dimensionless contribution to the relaxation modulus from each probe, $G_{k}\left(t;M_{k}\right)/\rho RT$ in sample PBD10. We emphasize that each of these probes feels the influence of the entire distribution through constraint dynamics.

To convert these predictions to the frequency domain, it is useful to represent the modulus of the *k*-probe as an analytic relaxation spectrum

(18) $$G_{k}\left(t;M_{k}\right)=G_{N}^{0}\int_{0}^{\infty }\frac{h_{k}\left(\tau \right)}{\tau }\exp\left(-\frac{t}{\tau }\right)d\tau ,$$

where $h_{k}\left(\tau \right)$ is the BSW relaxation function [17,21,37], given by

(19) $$h_{k}\left(\tau \right)=\frac{\sum_{i=1}^{m}\tau^{\alpha_{i}}\left[\Gamma \left(-\alpha_{i},\frac{t}{\tau_{i}}\right)-\Gamma \left(-\alpha_{i},\frac{t}{\tau_{i-1}}\right)\right]\prod_{j=0}^{i-1}\tau_{j}^{\alpha_{j}-\alpha_{j+1}}}{\sum_{i=1}^{m}\frac{\tau_{i}^{\alpha_{i}}-\tau_{i-1}^{\alpha_{i}}}{\alpha_{i}}\prod_{j=0}^{i-1}\tau_{j}^{\alpha_{j}-\alpha_{j+1}}},$$

where *m* is the number of modes, and $\tau_{i}$ and $\alpha_{i}$ are corresponding time constants and power-law exponents, all of which are determined by fitting the $G_{k}\left(t;M_{k}\right)$ data. We run simulations for at least a time $t_{\min}$, such that $G\left(t_{\min}\right)∼0.01G\left(0\right)$, to have a smooth G(t) and obtain a good estimation of the parameters. Polymers with less than 50 average entanglements fulfill this condition in a few simulation hours, but a simulation for chains with 500 entanglements takes a few months. This calculation depends on the computation resources.

Once we have an analytic expression for every $G_{k}\left(t;M_{k}\right)$ in a BSW form, we take the Laplace–Fourier transform to obtain the complex or dynamic modulus defined by

(20) $$G_{k}^{*}\left(\omega ;M_{k}\right)=i\omega {\hat{G}}_{k}\left[\omega ;M_{k}\right],$$

where the hat represent Laplace–Fourier transform. From this expression, we know the storage, $G^{\prime }$, and loss, $G^{\prime \prime }$, moduli for the *k*-probe as the real and imaginary parts of G*,

(21) $$G_{k}^{*}\left(\omega ;M_{k}\right)=G_{k}^{\prime }\left(\omega ,M_{k}\right)+iG_{k}^{\prime \prime }\left(\omega ;M_{k}\right).$$

Finally, we sum the contributions of all *k*-probes to $G^{*}\left(\omega \right)$, taking into account that every $G_{k}^{*}\left(\omega ,M_{k}\right)$ is weighted by a $W_{k}$ taken from the the weight distribution $W(M_{k})$

(22) $$\frac{G^{*}\left(\omega \right)}{\rho RT}=\int_{0}^{\infty }\frac{MG^{*}\left(\omega ,M\right)}{\rho RT}W\left(M\right)d\ln M.$$

In other words, we approximate the above integral using a cubic spline interpolation between the discrete probe contributions.

### 4.3. Results for the DSM

Again, we first consider PBD, but now starting with PBD20, which is a monodisperse melt with $PDI_{1}=1.1$. The simulation provides G* as a function of dimensionless frequency $\tau_{c}\omega$. Existence of a monodisperse sample is particularly useful for DSM for two reasons. First, the parameters are particularly easy to estimate. In addition, we have derived analytic expressions for the dynamic modulus, which can be used to fit the parameters precisely [21].

**Figure 7:**
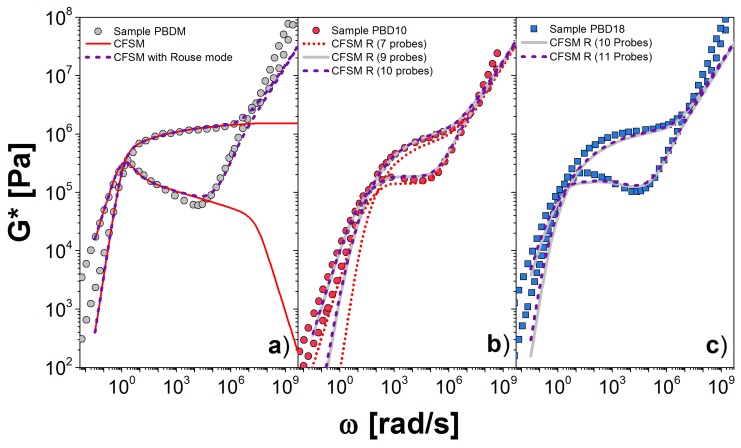
Dynamic moduli for PBD for: (**a**) monodisperse and polydisperse linear chains; (**b**) PBD10; and (**c**) PBD18 at 30 ${}^{\circ }$C. Symbols are the experimental results and lines the theoretical predictions. In (**a**), solid line corresponds to only the CFSM and dashed line to the CFSM including the Rouse modes. In (**b**,**c)**, we show the simulation data, including the Rouse modes, for polydisperse melts using different number of probes to represent the W(M) (see Figure 1a).

In Figure 7a, we present the comparison between the experiment (symbols) and the CFSM predictions, with and without Rouse modes, the solid and dashed lines, respectively. The theory provides a good estimation of G* over eight decades of frequency. At low frequency, there is a deviation in $G^{\prime }$ for ω∼0.1rad/s, which probably arises from the difficulty in extracting the storage modulus when the signal is dominated by the loss modulus. Alternatively, there might be some high-molecular-weight contaminant. At high frequency, there is also a clear deviation in both, $G^{\prime }$ and $G^{\prime \prime }$, for $\omega \geq 10^{8}$ rad/s, where glassy dynamics become important—physics that are neglected in the theory.

Figure 7b,c shows our calculation for the PBD10 and PBD18 samples, respectively, including the Rouse modes and using the $\tau_{c}$ values (and also β) obtained from the comparison with the monodisperse sample. The middle figure shows how the prediction changes as more (higher-molecular-weight) probes are added to the estimation. We see that for PBD10, 9 probes seem sufficient, but at least 10 or 11 are necessary for PBD18, in Figure 7c. The area shaded white in Figure 1 indicates the molecular weights covered by our simulation. We see that a significant amount of high-molecular-weight tail in PBD18 is not covered. Almost certainly, this discrepancy arises from our neglecting the higher molecular weight tail, as mentioned above. This tail could also be simulated, but is extremely expensive, since it involves calculations for polymers with averages of more than 700 entanglements.

**Figure 8:**
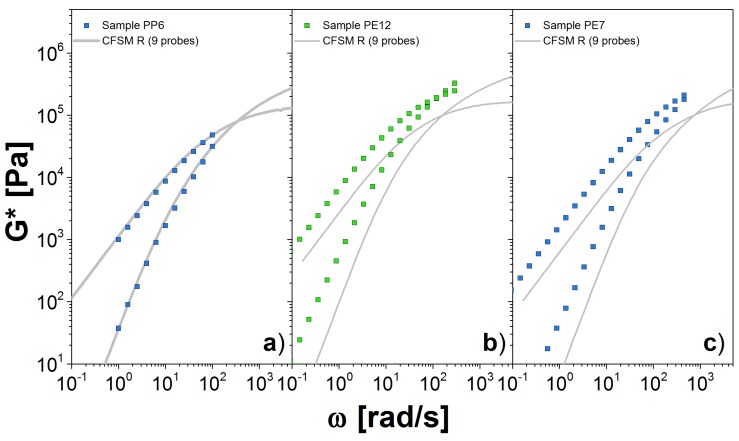
Dynamic moduli for polydisperse linear chains obtained through simulations using the slip-link model for (**a**) PP6 at 120 ${}^{\circ }$C, (**b**) PE12 at 190 ${}^{\circ }$C and (**c**) PE7 at 190 ${}^{\circ }$C. We use nine probes to represent the full distribution W(M). Symbols are the experimental results and lines the theoretical predictions.

Next, we simulate PP and PE samples. For those samples, we do not have a monodisperse system to determine the values of β and $\tau_{c}$. Instead, we use $G_{N}^{0(DRM)}$ to estimate β from Equations (14) and (15). Our chosen value for $\tau_{c}$ comes from a direct comparison with the dynamic modulus data. The parameters used for the theory are given in Table 3, and the predictions are shown in Figure 8.

For PP a reliable estimation of $G_{N}^{0}$ is approximately 1.0 MPa, which was obtained from the fitting of PP42. In general, we consider the value obtained for the larger molecular weight sample as the better estimation for $G_{N}^{0}$. In Figure 8a, we present the comparison between experiments and our theory simulations using nine probes. This number of probes allow us to cover most of the *M* values in W(M), so that the representation of the polydisperse system seems sufficient. Our prediction matches very well the experimental measurements in the entire ω range. Importantly, the plateau modulus coming from the DRM comparison seems to be a good estimator for the modulus needed as input in the CFSM. The simulations for samples PP28 and PP42 were avoided, again because we have to simulate highly entangled chains, otherwise we would obtain a poor estimate of G* as in the case of PBD18.

Finally, in Figure 8b,c, we show our results for the PE samples. The value for $G_{N}^{0}$ was found from DRM fit to PE12. For both cases, we computed the G* with only 9 probes, from the 10 probes that we need to correctly represent the W(M), that is because the calculation for the high molecular weight probe is computationally expensive. For PE12, we observe a reasonable agreement with the experimental measurements at low ω values. However, there are some differences for values close to the crossover point. Similar observations apply for PE7. It is possible that additional high-molecular-probes are necessary, but it seems more likely that the estimate for the plateau modulus obtained from DRM is not accurate. The unfortunate lack of any clear plateau for the PE data make a definitive conclusion on this point impossible.

## 5. Conclusions

We have modeled the dynamic moduli for polydisperse polymer melts using both a model based on double reptation (DRM), and the clustered fixed slip-link model (CFSM). Implementation of the DRM is inexpensive, and uses only two molecular weight-independent adjustable parameters, the plateau modulus ($G_{N}^{0}$) and a characteristic friction (*k*), which could be obtained from SAOS measurements, and the molecular weight distribution. The DRM makes several simplifying assumptions about polymer dynamics. However, from this model, we were able to fit G* for polymers with different molecular weight distributions. The agreement between this theoretical approach and experiments was good for polymers with similar $M_{w}$.

The CFSM requires analogous input: parameters that characterize the molecular weight distribution, as well as the entanglement activity β, and a characteristic time $\tau_{c}$. The β parameter is obtained from $G_{N}^{0}$; this quantity is most reliably obtained from rheological data for a monodisperse simple with high molecular weight, which is not available for every chemistry. Here, we show that a good estimation for β could be $G_{N}^{0(DRM)}$, which is obtained by using the DRM to study any polydisperse sample. The second parameter requires comparison with experimental data. We also attempted to calculate $\tau_{c}$ from $\tau_{0}$. However, such a calculation shows a variance of more than one order of magnitude, probably because of the large uncertainty in estimating $m_{p}$.

The CFSM is a much more expensive approach than the DRM. In particular, we found that we are limited computationally to chains less than about 500 entanglements. The computation for chains with around 700 entanglements is possible, but requires nearly two months to be done. However, such highly entangled chains are typically rather polydisperse, so could be handled reasonably well by the cheaper double reptation model.

## Figures and Tables

**Table 1:** Properties of the polymer melts studied in this work. PP and PE melts were produced and characterized in the ExxonMobil facilities. Properties of the PBD samples were already reported by Ruymbeke et al. [23]. In addition, we include the plateau modulus, density and molar mass of a Kuhn step, as given in Ref. [24] for samples at similar temperatures.

Polymer Name	$M_{w}$	${\mathrm{PDI}}_{1}$	*T*	$G_{N}^{0}$	ρ	$M_{K}$
	(g/mol)		(${}^{\circ }$C)	(MPa)	(g/mL)	(g/mol)
PP6	0.67 $\times \;10^{5}$	2.02	120	0.47	0.791	183.4
PP28	2.80 $\times \;10^{5}$	2.05	120	0.47	0.791	183.4
PP42	4.28 $\times \;10^{5}$	1.98	120	0.47	0.791	183.4
PP42	4.28 $\times \;10^{5}$	1.98	80	0.48	0.825	183.4
PE7	0.75 $\times \;10^{5}$	2.20	190	2.6	0.785	150.4
PE12	1.20 $\times \;10^{5}$	2.27	190	2.6	0.785	150.4
PBD10	1.08 $\times \;10^{5}$	2.7	30	1.15	0.895	112.5
PBD18	1.85 $\times \;10^{5}$	2.1	30	1.15	0.895	112.5
PBD20	2.04 $\times \;10^{5}$	1.1	30	1.15	0.895	112.5

**Table 2:** GEX parameters to describe the W(M) of PBD, PP and PE melts analyzed here. We also show our estimation for the plateau modulus parameter $G_{N}^{0(DRM)}$, the parameter *k*, and the characteristic time $\tau_{0}$ for the double reptation model described in Ref. [25]. The * indicates our reference system for which we estimated the value of $G_{N}^{0}$ used in the DSM approach, as explained in the main text.

Polymer Code	*a*	*b*	$m_{p}$	$G_{N}^{0(\mathrm{DRM})}$	*k*	$\tau_{0}$
			(g/mol)	(MPa)	(s(mol/g)${}^{3.5}$)	(μs)
PP6	1.33 (3)	0.75 (1)	13.7 (9) $\times \;10^{3}$	0.497	3.99 $\times \;10^{-20}$	12.02
PP28	2.2 (2)	0.48 (4)	5 (3) $\times \;10^{3}$	0.918	4.54 $\times \;10^{-20}$	0.363
PP42 *	2.04 (8)	0.54 (2)	15 (4) $\times \;10^{3}$	1.000	4.51 $\times \;10^{-20}$	19.05
PP42 *	2.04 (8)	0.54 (2)	15 (4) $\times \;10^{3}$	1.025	59.42 $\times \;10^{-20}$	251.19
PE7	1.26 (4)	0.64 (1)	9.1 (9) $\times \;10^{3}$	1.89	0.958 $\times \;10^{-20}$	0.69
PE12 *	1.36 (2)	0.61 (1)	11.8 (6) $\times \;10^{3}$	1.44	1.02 $\times \;10^{-20}$	1.82
PBD10	1.48 (4)	0.66 (1)	9.3 (9) $\times \;10^{3}$	0.690	20.4 $\times \;10^{-20}$	15.90
PBD18 *	1.96 (4)	0.54 (1)	6.7 (8) $\times \;10^{3}$	0.948	14.4 $\times \;10^{-20}$	5.47
PBD20	-	-	-	-	-	-

**Table 3:** Parameters used for our stochastic dynamics for the different polymer melts. $M_{c}$ is calculated from Equations (12) and (13), while β is obtained from Equation (14). $M_{c}^{\max}$ corresponds to the heaviest polymer we could simulate whose weight is approximately 700 $M_{c}$. The computationally unreachable region for simulation is shown as the shaded area in Figure 1. $\tau_{c}$ is calculated from the direct comparison with experiments.

Polymer Code	$G_{N}^{0}$	$M_{c}$	$M_{c}^{\max}$	β	$\tau_{c}$
	(MPa)	(g/mol)	(g/mol)		(μs)
PBD10	1.26	791.302	5.54 $\times \;10^{5}$	12.6	0.4
PBD18	1.26	791.302	5.54 $\times \;10^{5}$	12.6	0.4
PBD20	1.26	791.302	–	12.6	0.4
PP6	1.00	1282.71	8.97 $\times \;10^{5}$	11.5	0.3
PE12	1.44	1089.12	7.62 $\times \;10^{5}$	11.9	0.15
PE7	1.44	1089.12	7.62 $\times \;10^{5}$	11.9	0.15

## Abbreviations

DSM	discrete slip-link model
DRM	double-reptation model
PBD	polybutadiene
PP	polypropilene
PE	polyethylene
GEX	generalized exponential
PDI	polydisperse index
GPC	gel permeation chromatography
SAOS	small amplitude oscillatory shear
MWD	molecular weight distribution
CP	crossover point
CD	constraint dynamics
SD	sliding dynamics
CFSM	cluster fixed slip-link model
